# Gait and postural disorders in parkinsonism: a clinical approach

**DOI:** 10.1007/s00415-019-09382-1

**Published:** 2019-05-22

**Authors:** Cecilia Raccagni, Jorik Nonnekes, Bastiaan R. Bloem, Marina Peball, Christian Boehme, Klaus Seppi, Gregor K. Wenning

**Affiliations:** 1 Department of Neurology, Medical University of Innsbruck, Anichstrasse 35, 6020 Innsbruck, Austria; 2 Department of Neurology, Donders Institute for Brain, Cognition and Behavior, Radboud University Nijmegen Medical Centre, Nijmegen, The Netherlands; 3 Department of Rehabilitation, Donders Institute for Brain, Cognition and Behavior, Radboud University Nijmegen Medical Centre, Nijmegen, The Netherlands

**Keywords:** Axial disorders, Vascular parkinsonism, Multiple system atrophy, Progressive supranuclear palsy, Corticobasal syndrome

## Abstract

Disturbances of balance, gait and posture are a hallmark of parkinsonian syndromes. Recognition of these axial features can provide important and often early clues to the nature of the underlying disorder, and, therefore, help to disentangle Parkinson’s disease from vascular parkinsonism and various forms of atypical parkinsonism, including multiple system atrophy, progressive supranuclear palsy, and corticobasal syndrome. Careful assessment of axial features is also essential for initiating appropriate treatment strategies and for documenting the outcome of such interventions. In this article, we provide an overview of balance, gait and postural impairment in parkinsonian disorders, focusing on differential diagnostic aspects.

**Electronic supplementary material:**

The online version of this article (10.1007/s00415-019-09382-1) contains supplementary material, which is available to authorized users.

## Introduction

Gait disturbances, balance and postural impairments represent core axial symptoms of all parkinsonian syndromes. They lead to a loss of self-efficacy, a considerably reduced quality of life, falls and subsequent injuries as an inevitable consequence. Every clinical examination in patients with parkinsonism is incomplete without a careful evaluation of axial symptoms. As James Parkinson already noted in 1817, “…observation of patients begins while they are walking into the office” [1]. A recent paper emphasized the many clinical features that can be identified immediately when patients walk from the examination room towards the clinician’s office [2]. For example, important information can even be gained from observing how patients rise from the chair in the waiting room, from listening to the step cadence or from a shuffling sound due to reduced foot clearance [2]. However, the workup of axial disorders in clinical routine is often underestimated, incomplete or not performed properly. There are various reasons for this, including time constraints in busy clinics, or the small examination rooms where an accurate assessment of axial features is simply not feasible. However, to neglect gait and postural disorders in daily clinical practice is a missed opportunity, as these signs offer important clues to the nature of the underlying disorder, thus facilitating differential diagnosis.

Many different classifications of gait, balance and postural disorders have been suggested, pointing towards the importance of these signs in the clinical workup. A recent viewpoint [3] proposed a “sign-based approach”, whereby gait, balance and postural abnormalities represent the initial moment for a search into the underlying neurological syndrome, including Parkinson’s disease (PD), vascular parkinsonism (VP) and the atypical parkinsonian disorders (APDs) such as multiple system atrophy (MSA), progressive supranuclear palsy (PSP) or corticobasal syndrome (CBS). According to this sign-based approach, the clinical examination begins by observing patients in a seated position, then as they rise from a chair, followed by full standing and finally, while walking [3]. During this evaluation, the examiner pays specific attention to the various axial signs, which in many cases are fairly specific and can assist in making the differential diagnosis. A definite diagnosis can only be achieved by post-mortem examination. However, a careful medical history and a complete clinical examination, supported by accurate observation of axial patterns, may already lead the examiner to the right path during the patient’s life. Importantly, an early and reliable recognition of parkinsonian disorders is crucial with respect to proper counseling and patient management. For example, PD patients facing axial disability will typically adjust their movement pattern, aiming to move slowly and as safely as possible to avoid falls and injuries. In contrast, patients with PSP can manifest reckless behavior despite marked gait disability or postural instability. Timely recognition of this ‘motor recklessness’ can have clear implications for the rehabilitation approach, e.g., by restraining unsupervised mobility in PSP patients. The aim of this article is to provide an overview of characteristic gait, balance and postural disorders in PD versus VP and the various APDs, based on our own clinical experience and supplemented with existing literature. In illustrating the specific axial signs of parkinsonian syndromes, we will follow the sign-based approach described above [3]. Representative videos of axial disorders in VP, MSA, PSP and CBS are provided in the supplementary material.

## Parkinson’s disease

For PD patients, the typical profile of axial disturbance, as well as its evolution in the disease course are well established. We briefly illustrate these principal hallmarks here, as a basis for the comparison to patients with APD and VP. A change in gait pattern may be one of the earliest signs of PD, presenting with a slightly reduced arm swing on one side or with a subtle narrowing of the base of support [4]. However, axial disturbances in early stages of PD may not be immediately evident to the untrained eye, only becoming more visible in the middle-late stages (Hoehn and Yahr 3–4) [5], with a complex U-shaped relation with falls (these tend to peak in moderately affected patients, and then taper off again towards more severe disease stages when patients become progressively immobilized) [6].

While sitting, patients with PD may show an abnormal forward flexion of their posture across the entire body axis, including the trunk, neck and extremities [7]. Lateral trunk flexion is very common, occurring in up to 80% of patients [8, 9]. In late stages or in patients treated with antiparkinsonian drugs, rigidity of the extremities is often less pronounced than rigidity of the trunk [7].

While rising from a chair, PD patients are usually slow and may need more than one attempt or need their arms to rise [3]. This sit-to-stand difficulty can even occur in early disease stages, as reflected by a study that showed sit-to-stand difficulties in six patients with early PD (Hoehn and Yahr mean 1.75, range 1.5–2) [10].

During thoroughly standing (with eyes open), PD patients typically show a normal to narrow base of support, unlike most other neurological conditions [3]. This narrow-based stance becomes even more pronounced with disease progression [11, 12].

While walking, one of the earliest gait signs is an asymmetrically reduced arm swing amplitude [13]. The typical gait pattern of PD patients is again narrow based. Indeed, patients with early PD show no instability in the mediolateral plane and can typically perform the tandem gait test without taking any side steps [14, 15]. The presence of an abnormal tandem gait test (taking even a single side step when asked to make ten consecutive tandem steps is already considered as abnormal) in a PD patient at an early disease stage should be carefully evaluated, as it may suggest an underlying APD, or occasionally the presence of cerebrovascular co-morbidity in patients with otherwise typical PD. With further disease progression, instability in the mediolateral plane may occur, as reflected by an abnormal tandem gait test, but this seems mainly related to development of cerebrovascular comorbidities rather than to PD itself [14]. Notably, PD patients can still ride a bicycle until late stages of the disease, as reported by a prospective, observational study in 156 patients with parkinsonism which showed that a form of APD should be considered when patients spontaneously stop riding a bicycle because they feel too insecure [16].

In accordance, 88% (*n* = 16) of all patients in the EMSA-PIGD study (details are shown below) had a normal tandem gait test, while only 12% (*n* = 3) exhibited a pathological timed up and go test (TUG-test). The vast majority (83%) had no postural instability in the retropulsion test [15].

In advanced stages of PD, gait disturbances become more complex and start to represent a major determinant of disability and poor quality of life [17, 18]. Optimized drug treatment or deep-brain stimulation are usually not sufficient to control gait and balance adequately. Moreover, cognitive decline worsens which can in turn negatively influence gait [19]. Freezing of gait (FoG) now becomes a common finding. FoG is defined as a “brief, episodic absence or marked reduction of forward progression of the feet despite the intention to walk” [20], also including episodes of “start hesitation” or shuffling gait with steps that range from millimeters to a couple of centimeters. It is one of the most debilitating symptoms of PD. FoG is usually most common and most pronounced during the “off” phases in late stages of PD, although it can also occur with briefer episodes during the “on” phases [21]. An estimated 25–60% of PD patients report episodes of FoG [22–26], but the actual figures may well be higher given the difficulties to detect FoG in the clinic [27, 28].

Most commonly, FoG occurs when patients are starting to walk, turning, going through narrow passages or approaching a destination [29]. Although less common, it can also occur while patients are walking straight ahead, namely when environmental or emotional situations occur such as unexpected events that raise arousal [29]. Due to its episodic nature, FoG is notoriously difficult to provoke in clinical practice. Asking patients to make rapid full turns “on the spot” (and in both directions) is the best way to provoke FoG [28, 30]. This test should always be applied, even when gait looks fine otherwise.

Festination is one of the most characteristic features of PD-gait. Clinically, it is defined as the tendency to move forwards with increasingly fast and small steps, and it is associated with the center of gravity falling forwards over the stepping feet [29]. It is often accompanied by FoG, co-occurring in the same patient, and is also typical for later disease stages. Recently, another phenotype of festination has been described, distinguished by a forward leaning of the trunk due to an impairment in postural control associated with ineffective small-balance correcting steps, as such being an expression of a balance control deficit [31].

## Vascular parkinsonism

VP, also known as lower body parkinsonism, is dominated by postural instability and a broad-based gait disorder caused by vascular white matter lesions in patients with a vascular risk profile. VP accounts for 4.4–12% of all cases of parkinsonism [32]. It was first described by Critchley et al. [33], who coined the term “arteriosclerotic parkinsonism” and, about 60 years later, was renamed as “lower half parkinsonism” by Thompson and Marsden [34]. This latter term emphasizes its clinical hallmarks that substantially differ from PD regarding the relative absence of upper limb akinesia and the early predominance of postural instability and a broad-based gait [35].

While sitting, patients with VP show relatively less symptoms compared to patients with PD [35] where upper limb signs (tremor, rigidity or slowness) are more prominent. Rest tremor was observed in only 4% of VP patients versus 47% of PD patients [36]. Postural tremor seems more common in VP [37].

When rising from a chair, VP patients almost invariably show a widened base and postural instability represents an early symptom [35]. A broad-based, unsteady stance is a sensitive but non-specific sign during full standing [3]. The typical postural abnormality of VP is characterized by a fairly upright posture without flexion, and straight legs with extension of the knees and hips [34].

Gait disturbance is an overwhelming clinical aspect of VP and differs substantially from PD. One study reported that 90% of VP patients present with a disproportionate gait impairment in initial stages (disease duration 2.6 ± 1.5 years), in contrast to only 7% of PD patients matched for disease duration [38]. Gait is unsteady, broad based and start hesitations are common, steps are shuffling, with possible reduced bilateral arm swing, resulting in a “stiff, wooden appearance, with loss of the normal synergy, fluency and dynamic interplay of arms, trunk, and legs” [34] (online resources 1). Nevertheless, a quantitative gait analysis study of 12 patients with VP, 12 patients with PD and 10 healthy controls demonstrated a relatively preserved arm swing in VP patients [39], thus we find this a useful feature for the differential diagnosis from other parkinsonian syndromes. Turning and walking “en bloc” is also a notable feature of VP, mainly reflecting axial immobility. Specifically, VP patients show a limited sideways turning of the head, neck and shoulder [35]. A subtler sign is the outward rotation of the feet during walking, suggesting underlying balance impairment. The broad base of support together with a variable step length looks ataxic, although in this case more of a frontal ataxia than a cerebellar ataxia [35] (see online resource 1). Throughout the disease course, postural instability and falls become more pronounced, representing a transition to wheelchair dependency and immobility [34, 35]. FoG is also a common feature of VP. Giladi et al. [40] conducted a database survey of FoG occurrence in 347 patients with a parkinsonian syndrome other than PD, and found FoG in 25 out of 44 patients with VP (57%). Even the FoG episodes tend to be broad based in VP patients, unlike the more narrow-based FoG episodes in PD patients. Festination has also been described in VP [3].

## Multiple system atrophy

MSA is a rapidly progressive neurodegenerative disease characterized by any combination of autonomic, parkinsonian, cerebellar, and pyramidal features [41]. Two motor phenotypes are recognized: a parkinsonian variant (MSA-P) and a cerebellar variant (MSA-C), both of them being commonly associated with symptoms of autonomic failure.

While sitting, MSA patients may show a tendency to lean sideways, due to a pronounced tonic lateral flexion of the trunk termed Pisa syndrome [42]. Furthermore, MSA patients can exhibit a marked forward flexion of the neck (disproportionate antecollis) (see online resource 2) [43], which is out of proportion to the degree of anteflexion in other body regions [44]. Look-alikes of such an antecollis can also be found in other neurological conditions (e.g., myasthenia gravis, polymyositis, amyotrophic lateral sclerosis), even though the antecollis is typically weak or flaccid, whereas the antecollis in MSA is stiff and frequently barely mobile [3]. When seen in parkinsonian patients, a disproportionate antecollis is considered to be a “red flag” that signals the possible presence of MSA [45].

MSA patients usually show a broad-based stance after rising from a chair and during thoroughly standing. Such postural changes from a supine/sitting to a standing position may aggravate orthostatic hypotension, a key feature of MSA [46-48] that leads to dizziness or outright syncope, thereby playing an important role in the risk of falls [48, 49].

Patients with MSA usually show a broad-based gait consistent with midline cerebellar ataxia that commonly occurs in MSA-C, but also in MSA-P [44]. This gait pattern differs strikingly from PD patients that show a narrow-based gait. Broad-based gait in MSA represents a consequence of their instability in the mediolateral plane, which is likely related to the more widespread underlying neuropathology, extending beyond the nigrostriatal pathway and also including the cerebellum, brainstem and their connections [3]. This can be unveiled by the tandem gait test [14, 15], which has been found to be abnormal in 85% of 21 MSA patients in the EMSA-PIGD study. In the same study, 56% of patients had postural instability in the retropulsion test and 60% performed a pathological TUG-Test [15]. Mediolateral instability is also reflected by the inability of MSA patients to ride a bike.

The prevalence of FoG was initially reported to be low in patients with MSA [50], but a recent publication showed that a high proportion (75%, study population 28 patients) of ambulatory MSA patients with a mean disease duration of 6.4 ± 4.0 years report FoG, both in MSA-P (82%) and MSA-C (50%). In this study, FoG appeared more frequently in advanced stages of MSA-C but showed no correlation to disease duration in MSA-P [50], suggesting an association with lesions in the basal ganglia rather than the brainstem or cerebellum. These data are in line with an analysis of FoG in post-mortem-confirmed MSA, which also identified a FoG rate of 40% at first (median 36 months after symptom onset) and 54% at last visit [50, 51]. Therefore, the occurrence of FoG in MSA should always be explored carefully. Unlike PD, the arm swing reduction in MSA is usually more symmetrical. Furthermore, due to involvement of the autonomic nervous system, patients with MSA tend to have frequent falls caused by syncope resulting from neurogenic orthostatic hypotension [48].

## Progressive supranuclear palsy

The classical phenotype of PSP is the Richardson’s syndrome (PSP-RS), which is characterized by vertical supranuclear gaze palsy (definitive diagnostic feature) in combination with other symptoms (e.g., bradykinesia, subtle personality changes, unexplained falls, bradyphrenia, executive dysfunction, eyelid apraxia, dysarthria) [52]. According to predominant clinical features, other PSP subtypes have been described [52]. Among these, the primary progressive freezing gait is of particular interest, presenting with a progressive neurological disorder which primarily affects gait, beginning with freezing and resulting later in postural instability [53].

While sitting, patients with PSP tend to show a rather characteristic backward dropping of the head (retrocollis) [3, 54], although it can also be seen occasionally in patients with recessive PD. In the classical phenotype, the gaze is wide eyed and the eye blink frequency is reduced [55]. More specifically is the impairment of downward gaze in PSP patients (online resource 3) [55]. Another specific feature of PSP patients is the frontal lobe disturbance, leading to signs such as the so-called “rocket sign” [3], which is notable when patients are asked to rise from a chair. Here, when attempting to stand up, PSP patients may rise far too rapidly given their degree of postural instability, only to topple backwards into their chair.

During full standing, upright stance is commonly impaired, leading to a gradual backward drift [56]. Even small challenges by sudden internal or external perturbations can lead to marked instability [57]. Base of support is usually broad.

The gait of PSP patients has been described as a “drunken sailor”-like gait [55], where prominent postural instability and frequent falls within the first year of disease onset are typical features (online resource 4) [58]. Notably, data from the EMSA-PIGD also included 25 PSP-RS patients and has shown postural instability in the retropulsion test in 72%, an abnormal TUG-test in 71% and a tandem gait test with side steps in 70% of these PSP patients [15].

A peculiar phenomenon of PSP is the “careless walking” or “motor recklessness”, which often leads to “reckless falls”, caused by a deficient risk estimation and lack of attention. Patients move too quickly, with abrupt stops and turns, as if they ignore their prominent balance deficits [20]. Tripping by walking is also common and it is due to the inability to veer gaze downward [55]. Similar to MSA, PSP patients show a broad-based gait [59]. Recent data about FoG indicate a high prevalence in PSP. Specifically, a recent study followed 401 patients (40 with PSP, mean disease duration of 3 months and 361 with other neurodegenerative disorders) for at least 1 year and reviewed symptoms and signs in a standardized manner, considering vertical supranuclear gaze abnormality and movement disorders including FoG as clinical core features. In this study, FoG was an early symptom of PSP and even occurred before a vertical supranuclear gaze abnormality emerged, thus improving a timely clinical diagnosis [60].

## Corticobasal syndrome

CBS and its pathological entity corticobasal degeneration (CBD) are rare neurodegenerative diseases [61]. The term CBD refers to the deposition of abnormally hyperphosphorylated microtubule-associated tau protein in the somatosensory, premotor and supplementary motor cortices, as well as the brainstem and basal ganglia [62]. In most cases, CBS is a tauopathy but the disease is not always due to corticobasal degeneration, suggesting some clinicopathological heterogeneity [63]. The clinical syndrome of CBS is complex, characterized by limb clumsiness, marked asymmetrical parkinsonism-dystonia, apraxia, cortical deficits, myoclonus and dementia in various combinations [61]. Usually, CBS presents with an asymmetric progressive ideomotor apraxia involving the upper limb and associated with rigidity, myoclonus, and dystonia [55]. At early disease stages, the upper extremities are more frequently affected. During the disease course, the lower extremities become affected as well.

While sitting, patients with CBS usually show a marked asymmetry in motor features, such as dystonia, rigidity, myoclonus, or tremor [64]. One of the most crucial signs seems to be the “useless arm”, with or without alien limb phenomena (online resource 5) [64].

While rising from a chair, disequilibrium and broad base of support seem to be very common in early disease stages, as reported by Rinne et al. [65], who analyzed the clinical features of 36 patients with pathologically proven or clinically probable CBS. In the latter study, within 3 years, 19 out of 36 patients presented with apraxia of gait, leg stiffness interfering with walking, and subsequently with shuffling gait, reduced stride length, and start or turn hesitations, suggesting an underlying frontal gait disorder [66]. Five patients reported general unsteadiness and postural impairment, due to subcortical disequilibrium. Of interest, patients who presented with gait disturbance as an early feature had a dramatic disease course, using a wheelchair after a median of 2 years.

A natural history study of pathologically confirmed CBS patients [67] supported the observations of Rinne et al. Here, gait disturbance and postural instability were present in 36% of patients, with falls presenting within the first year of onset in 21% of patients. Gait disorders were almost always associated with postural instability and characterized by small steps, unstable and apraxic gait, bradykinesia, and a widened base. As for other APDs, wheelchair dependence is an inevitable outcome in these patients. FoG can also be present in CBS. However, its prevalence seems to be lower compared to MSA and PSP in early disease stages. As reported by Mueller et al., who examined the frequency of FoG in 13 pathologically confirmed cases of CBD, 8% of CBD patients showed FoG at the first visit, while 25% showed it at the last visit, 5 months before death [21, 51].

## The EMSA-PIGD study: gait and balance tests discriminate PD and APD

Additional tests can be used to elicit informative gait and postural impairment. As indicated earlier, symptoms of postural instability and gait disability (PIGD) represent a core feature of APD and VP patients. Clinical assessment of PIGD symptoms is not standardized and clinicians often explore PIGD symptoms using a variety of tests that may be time consuming or diagnostically superfluous [15]. To address this aspect, a recent multicenter cohort study evaluated the diagnostic value of a battery of widely used PIGD tests, with the aim of developing a brief PIGD evaluation that can elicit additional information about gait and postural impairment and, therefore, help to differentiate PD from APD patients [15]. 19 PD patients, 21 MSA-P and 25 PSP-RS with a mean of 4 years of disease duration were included in 11 European MSA Study (EMSA) sites and were enrolled prospectively over a period of 2 years. The data showed that the best discriminative power was yielded by the combination of timed up and go test (TUG-test, AUC 0.77; 95% CI 0.64–0.9; *p* = 0.001), tandem gait (AUC 0.83; 95% CI 71–94; *p* < 0.001) and retropulsion test (AUC 0.8; 95% CI 0.69–0.91; *p* < 0.001), thus providing a standardized “bedside” test battery for the assessment of PIGD symptoms. The composition of this battery is grounded in the fact that each specific test investigates different systems: the TUG-test exploring arising from a chair, walking and turning; the tandem gait test evaluating stability in the mediolateral plane; and the retropulsion test evaluating stability in the anterior–posterior plane. For the TUG-test, a cutoff of 16 s discriminated between pathological and normal performances. Despite yielding a smaller discriminative value than the tandem gait, the “bicycle sign” was found to be complementary to the tandem gait test, as it provides information about instability in the mediolateral plane. A re-evaluation of the diagnosis should, therefore, be considered when patients previously diagnosed as having PD indicate that they stopped riding a bicycle because of self-perceived instability.

## Conclusion

**Fig. 1:**
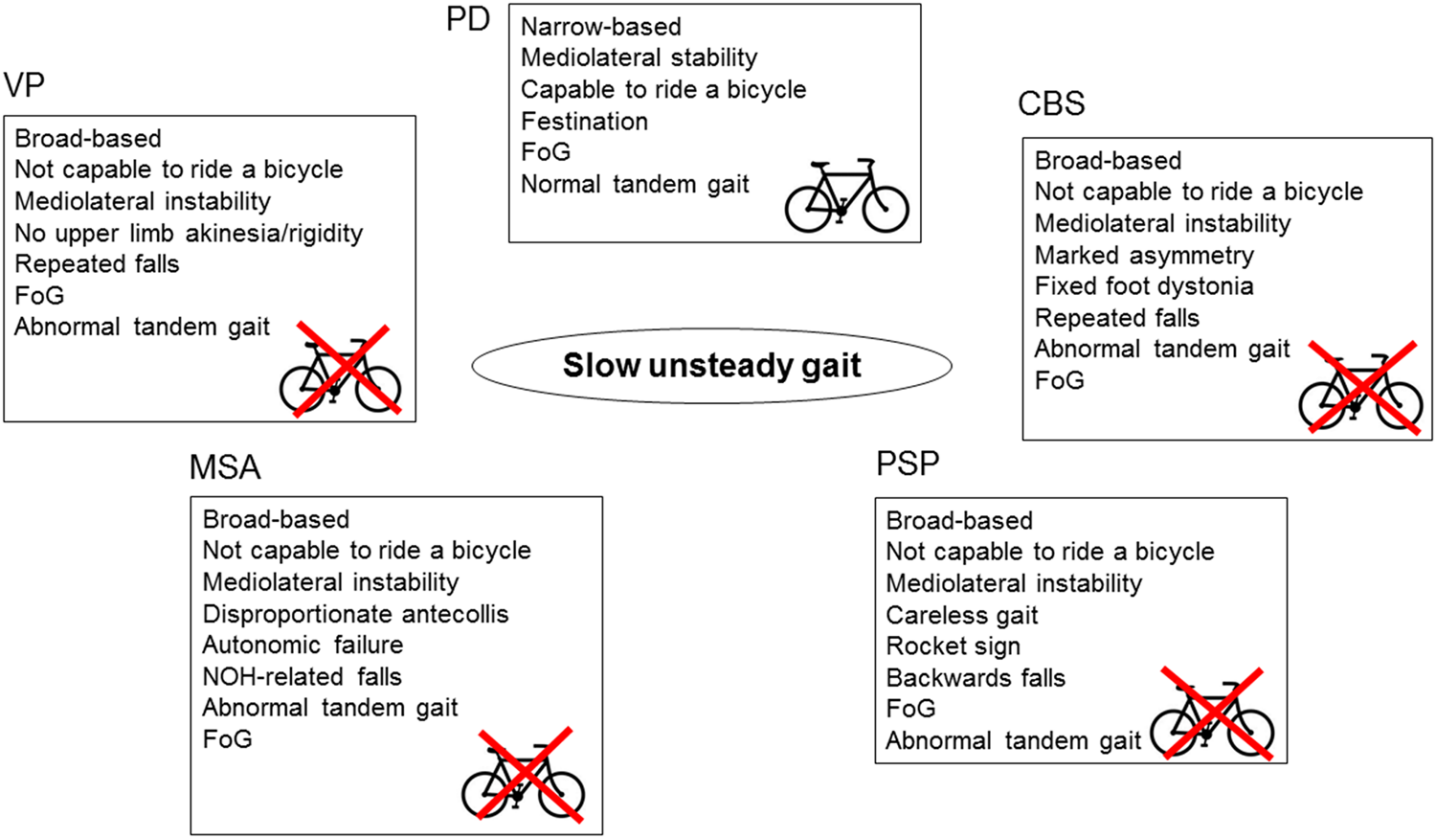
Axial features in parkinsonian disorders. *PD* Parkinson’s disease, *VP* vascular parkinsonism, *MSA* multiple system atrophy, *PSP* progressive supranuclear palsy, *CBS* corticobasal syndrome, *FoG* freezing of gait, *NOH* neurogenic orthostatic hypotension. Microsoft PowerPoint 14.0.7229.5000 (2010) and Adobe Photoshop CS6 13.0 x32 were used to create this figure

The axial hallmarks of PD, MSA, PSP and VP are summarized in the figure (Fig. 1). Although an accurate neurological diagnosis in the absence of post-mortem verification cannot be achieved in any of these disorders, axial features may contribute significantly to the diagnostic differentiation. Axial signs such as a broad base of support, an abnormal tandem gait or inability to ride a bike, or the presence of recurrent falls in early disease stages should alert the neurologist and lead to the differential diagnosis of non-PD disorders. Additional warning signs (“red flags”) can further contribute to a correct clinical diagnosis, i.e. lower half parkinsonism for VP; early-onset severe autonomic failure, ataxia, disproportionate antecollis, orofacial dystonia, and stridor for MSA; frontal lobe impairment, motor recklessness, recurrent falls and vertical supranuclear gaze palsy for PSP; and limb dystonia, apraxia and marked asymmetry for CBS. The combination of the TUG-test, tandem gait test, and retropulsion test is the most sensitive battery to discriminate PD from APD. Future studies remain needed to further validate this test battery.

## Electronic supplementary material

Below is the link to the electronic supplementary material.

Supplementary file1 (MP4 19789 kb) ESM_1_VP This video shows a patient with ischemic lesions of the basal ganglia (caudate nucleus, putamen) on the right side with a slow, broad-based, ataxic, spastic gait disorder

Supplementary file2 (MP4 23782 kb) ESM_2_MSA This video shows a patient with MSA-P with an unsteady slow gait as well as freezing of gait while turning. The postural stability is severely reduced

Supplementary file3 (MOV 46380 kb) ESM_3_PSP_eyes This video shows a PSP patient with saccadic eye movements in the horizontal plane and an impairment in vertical gaze with reduced amplitude

Supplementary file4 (MP4 25697 kb) ESM_4_PSP This video shows a patient with PSP with a broad-based, instable gait and difficulties while turning. The postural reflexes are reduced

Supplementary file5 (MP4 58344 kb) ESM_5_CBS This patient shows severe apraxia of both hands (left>right) in a patient with CBS

Supplementary file6 (MP4 22901 kb) ESM_6_CBS This patient with an early CBS shows severe apraxia and dystonia, mainly of the left hand and a stiff gait
